# Spatially Varying *Wolbachia* Frequencies Reveal the Invasion Origin of an Agricultural Pest Recently Introduced From Europe to North America

**DOI:** 10.1111/eva.70016

**Published:** 2024-09-20

**Authors:** Sonja Lečić, Thomas M. Wolfe, Animesh Ghosh, Serdar Satar, Camilla Souza Beraldo, Emily Smith, Jason J. Dombroskie, Emily Jernigan, Glen Ray Hood, Hannes Schuler, Christian Stauffer

**Affiliations:** ^1^ Department of Forest and Soil Sciences Boku University Vienna Austria; ^2^ Department of Plant Protection, Faculty of Agriculture Çukurova University Adana Turkey; ^3^ Organismal and Evolutionary Biology Research Programme, Faculty of Biological and Environmental Sciences The University of Helsinki Helsinki Finland; ^4^ Department of Biological Sciences Wayne State University Detroit Michigan USA; ^5^ Department of Entomology Cornell University Ithaca New York USA; ^6^ Competence Centre for Plant Health Free University of Bozen‐Bolzano Bozen‐Bolzano Italy; ^7^ Faculty of Agricultural, Environmental and Food Sciences Free University of Bozen‐Bolzano Bozen‐Bolzano Italy

**Keywords:** endosymbiont, European cherry fruit fly, invasive species, *Rhagoletis cerasi*, spatial interpolation

## Abstract

The introduction of non‐native species across the world represents a major global challenge. Retracing invasion origin is an important first step in understanding the invasion process, often requiring detailed sampling within the native range. Insect species frequently host *Wolbachia*, a widespread endosymbiotic bacterium that manipulates host reproduction to increase infected female fitness. Here, we draw on the spatial variation in infection frequencies of an actively spreading *Wolbachia* strain *w*Cer2 to investigate the invasion origin of the European cherry fruit fly, *Rhagoletis cerasi.* This pest of cherries was introduced from Europe to North America within the last decade. First, we screen the introduced fly population for the presence of *Wolbachia*. The introduced populations lack the *w*Cer2 strain and the strongly associated mitochondrial haplotype, suggesting strain absence due to founder effects with invading individuals originating from *w*Cer2‐uninfected native population(s). To narrow down geographic regions of invasion origin, we perform spatial interpolation of the *w*Cer2 infection frequency across the native range and predict the infection frequency in unsampled regions. For this, we use an extensive dataset of *R. cerasi* infection covering 238 populations across Europe over 25 years, complemented with 14 additional populations analyzed for this study. We find that *R. cerasi* was unlikely introduced from *w*Cer2‐infected populations in Central and Western Europe. We propose *w*Cer2‐uninfected populations from Eastern Europe and the Mediterranean region as the most likely candidates for the invasion origin. This work utilizes *Wolbachia* as an indirect instrument to provide insights into the invasion source of *R. cerasi* in North America, revealing yet another application for this multifaceted heritable endosymbiont. Given the prevalence of biological invasions, rapidly uncovering invasion origins gives fundamental insights into how invasive species adapt to new environments.

## 1 Introduction

Facilitated by increasing global connectivity, biological invasions are integral to global environmental change (Early et al. 2016; Pyšek et al. 2020). The growing concerns have steered researchers toward a better understanding of how species adapt to new environments (White et al. 2013; Tepolt et al. 2022) and disentangling the factors that enable their range expansions (Deshpande and Fronhofer 2022). An essential first step in understanding biological invasions is to uncover the invasion origin of introduced species (Dlugosch and Parker 2008; Estoup and Guillemaud 2010). Identifying the source populations relies on a careful and detailed sampling within the native range (Dlugosch et al. 2015) because demographic processes such as founder effect and admixture can confound the inference of invasion origins (Bock et al. 2015; Sherpa and Després 2021).

Due to founder effects, introduced populations may carry a fraction of the genetic diversity compared to populations in their native range (Prentis et al. 2008; Szűcs et al. 2017). Similarly, upon introduction, a fraction of invading individuals can bring their obligate residents into a new range (Hurst and Jiggins 2005; Lu, Hulcr, and Sun 2016) or experience a reduction in endosymbiont diversity (Reuter, Pedersen, and Keller 2005; Shoemaker et al. 2000). *Wolbachia*, a widespread bacterial endosymbiont among insects (Werren and Windsor 2000; Jiggins et al. 2001), occupies the germline of its insect hosts and is vertically inherited through the maternal line. A common way in which *Wolbachia* manipulate host reproduction and ensure a reliable transmission in the new host is through cytoplasmic incompatibility (CI) (Turelli and Hoffmann 1991). Here, uninfected females produce few or no offspring when mating with infected males or males infected with another *Wolbachia* strain (Hoffmann and Turelli, 1988).

The European cherry fruit fly, *Rhagoletis cerasi* (Linnaeus) (Diptera: Tephritidae), is a severe pest in cherry orchards (genus *Prunus*), but can also be found infesting honeysuckle berries (genus *Lonicera*). The fly is widespread across its native range in Eurasia (Boller and Prokopy 1976). All native populations of *R. cerasi* are fixed for a *Wolbachia* strain *w*Cer1. In addition, *R. cerasi* can host coinfection by a second strain, *w*Cer2. However, unlike the fixed *w*Cer1 strain, *w*Cer2 is found in spatially varying frequencies in *R. cerasi* populations across Europe, ranging from (near) fixed in some locations, but completely absent in others (Riegler and Stauffer 2002; Schuler et al. 2016; Bakovic et al. 2018; Schebeck et al. 2019). The *w*Cer2 strain causes strong CI between *w*Cer2‐infected males and *w*Cer2‐uninfected females, with laboratory estimates of CI as high as 98% (Boller et al. 1976; Riegler and Stauffer 2002). In the native range, *R. cerasi* harbors two mitochondrial haplotypes, denoted HT1 and HT2 (Schuler et al. 2016). The HT1 variant is associated with *w*Cer1‐infected flies, whereas mitochondrial haplotype HT2 is associated with the spreading and CI‐causing *w*Cer2 strain. In Central Europe, *w*Cer2 has spread in *R. cerasi* through CI, causing haplotype HT2 to hitch‐hike alongside the *Wolbachia* strain (Schuler et al. 2016). This sweep presumably replaced HT1 in the *w*Cer2‐infected populations, with deviations in transitional populations detected (Schuler et al. 2016; Schebeck et al. 2019).

Classical models of *Wolbachia* infection frequency dynamics consider the proportion of uninfected eggs produced by infected females (*μ*), the relative reproductive success of infected and uninfected females (F), and the strength of CI (H) as the three main factors affecting changes in infection frequency (Hoffmann, Turelli, and Harshman 1990). If initially very rare, *Wolbachia* frequency will either stabilize at 0% or must satisfy *F* (1–*μ*) > 1 to spread deterministically from low frequencies, regardless of whether they cause CI (Kriesner et al. 2013; Turelli, Katznelson, and Ginsberg 2022). Once sufficiently common, the effects of strong CI push *Wolbachia* to high‐equilibrium frequencies (Hoffmann, Turelli, and Harshman 1990; Barton and Turelli 2011). Reproductive manipulations with strong effects pave the way to the rapid spread of new invading *Wolbachia* strains, exemplified with *w*Ri in *Drosophila simulans* populations (Turelli and Hoffmann 1991, 1995; Kriesner et al. 2013). A fast spread can also reduce mitochondrial diversity or promote mitochondrial polymorphisms in host populations (Turelli and Hoffmann 1991; Turelli, Hoffmann, and McKechnie 1992; Jiggins and Tinsley 2005; Xiao et al. 2012; Arif et al. 2021). Such rapid spread creates geographical differences in the *Wolbachia* infection frequency and, sometimes, mitochondrial diversity through the fly's geographic range (Turelli and Hoffmann 1991; Hoshizaki and Shimada 1995; Schuler et al. 2016). Due to this spatial heterogeneity, we hypothesize that in natural conditions *Wolbachia* can serve as an indirect tool to infer insect invasion routes.

Recent reports indicate that *R. cerasi* was introduced into the northeastern United States and southeastern Canada sometime within the last decade (Barringer 2018; Wakie, Yee, and Neven 2018). In 2016, the fly was first found in Mississauga, Ontario, Canada, and again in 2017 in the United States in Niagara County, New York (Barringer 2018; Wakie, Yee, and Neven 2018) near or in stands of introduced European honeysuckle. The United States Department of Agriculture (USDA) now considers the fly a threat for farmers and stakeholders in the region where *R. cerasi* is currently under regulatory control, restricted to a quarantine area comprising five counties in western New York, covering a total of 5187 km^2^. To further underscore the broader implications of the threat of this pest species, environmental modeling suggests that the fly could spread to and establish in the major cherry‐growing states of Michigan, Washington, Oregon, and California where production is collectively valued at ~$700 million US annually (Wakie, Yee, and Neven 2018). Therefore, there is a need to control the spread of the fly, prevent future invasions, and elucidate the pathways of invasion from the fly's native range in Europe.

In this study, we take advantage of the differences in infection frequencies of the *w*Cer2 strain in *R. cerasi* across the native range to infer the region(s) from which the fly was introduced into North America. To corroborate the results gleaned from comparing the frequencies of the *w*Cer2 strain in the native in introduced population, we take advantage of the *w*Cer2 association with the mitochondrial haplotype HT2. The invasive population lacked the *w*Cer2 strain and the associated mitochondrial haplotype, suggesting that the fly likely originating from a *w*Cer2‐uninfected native population. We then combine infection frequency information from 14 populations sampled for this study with the *w*Cer2 infection frequency dataset consisting of an additional 238 populations sampled over the last 25 years across the fly's native range (Riegler and Stauffer 2002; Schuler et al. 2016; Bakovic et al. 2018), and perform spatial modeling to predict the invasion origin. Ultimately, our study highlights the utility of endosymbionts as a prospective tool for invasion genetics research and discuss our findings in light of *Wolbachia* and host mitochondria interactions.

## 2 Materials and Methods

### 2.1 Estimating *w*Cer2 Infection Frequencies and mtDNA Haplotyping

**FIGURE 1:**
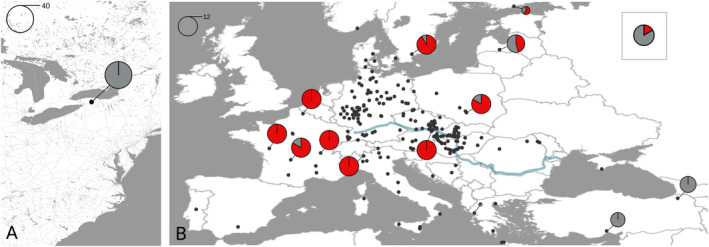
Geographic locations and *w*Cer2 infection frequencies of *Rhagoletis cerasi* populations and the distribution of *w*Cer2 in (A) the introduced range in Niagara County, New York state, in northeastern United States and (B) the native range across Europe. Due to a very high sampling density in some regions and large sample sizes for several populations, locations from prior studies are represented as single black dots. The infection frequencies of the 13 native populations including the population from Russia (inset) sampled in this study are represented with pie charts showing *w*Cer2 infection frequency (red: *w*Cer2‐infected; grey: *w*Cer2‐uninfected). The size of each pie chart reflects the number of individuals sampled at each location.

To determine the infection frequency of the *w*Cer2 strain in the introduced range, *R. cerasi* adult flies were caught on yellow sticky traps from several locations in Niagara County, collectively referred to as a single population within the introduced range in 2018 (Figure 1A; Table S1). Adult flies were removed from the sticky traps, cleaned using histoclear, identified to species using distinguishing characteristics in wing banding patterns, sexed, and stored in 70% ethanol. The DNA of whole‐body individuals was extracted using the Qiagen DNeasy Blood and Tissue extraction kit following the manufacturer's protocol.

To estimate distance‐dependent population variance of the *w*Cer2 infection frequency in the native range, we sampled additional *R. cerasi* populations from *Prunus* trees in 14 locations between 2018 and 2022 (Figure 1A; Table S1). Our motivation for the choice of sampling locations was twofold. First, we resampled several regions of the native range to test for patterns of temporal stability in *w*Cer2 frequencies. In this case, observations of a general lack of temporal variation in the uninfected and fixed *w*Cer2 populations would enable us to pool all field estimates of the *w*Cer2 infection frequencies spanning 25 years and perform a spatial population variance analysis to predict infection frequencies in unobserved locations. Second, to improve the accuracy of distance‐dependent population variance estimates of the *w*Cer2 infection frequency, we sampled populations from Turkey, Finland, Sweden, Latvia, and Russia. These populations represent the first field estimates of *w*Cer2 infection frequencies from these regions of the native fly range.

To sample flies in the native range, we collected and placed cherry fruits sampled at each location in plastic trays at room temperature and stored emerging larvae upon pupation in 95% ethanol. The total DNA of single pupae was extracted using the Qiagen Gentra Puregene extraction kit following the manufacturer's protocol. We then used PCR to assess the infection status (presence vs. absence) of each individual from each location in the native and introduced ranges following Schuler et al. (2016) with *w*Cer2‐specific primers targeting regions of the surface protein *wsp* (Arthofer et al. 2009). The proportion of infected flies served as our estimate of infection frequency (*p*) for each location. To score the presence versus absence of *w*Cer2 strain infection in the native and introduced populations, we visualized PCR products via electrophoretic separation on 2% ethidium bromide‐stained agarose gels. To distinguish between mitochondrial haplotype HT1 and HT2 for an individual sample (Table 1), we amplified, sequenced and scored the 546‐bp fragment of the cytochrome oxidase I (*COI*) gene using the primers following the methods of Schuler et al. (2016).

**TABLE 1:** Sample size (*N*), number of *w*Cer2‐infected individuals, infection frequencies *p* with exact 95% binomial confidence intervals, and mitochondrial haplotype are shown for each *Rhagoletis cerasi* population sampled from 2018 to 2020 in the native and introduced range.

Population	*N*	*w*Cer2‐infected	*p* (confidence intervals)	Haplotype
Austria	12	12	1 (0.758, 1)	HT2
Italy	12	12	1 (0.758, 1)	HT2
France 1	12	12	1 (0.758, 1)	HT2
France 2	12	10	0.833 (0.552, 0.953)	HT1
France 3	12	12	1 (0.758, 1)	HT2
France 4	12	12	1 (0.758, 1)	HT2
Turkey 1	9	0	0 (0, 0.299)	HT1
Turkey 2	10	0	0 (0, 0.278)	HT1
Russia	12	2	0.167 (0.0470, 0.448)	HT2
Greece	2	0	0 (0, 0.658)	HT1
Poland	12	10	0.833 (0.552, 0.953)	HT1, HT2
Sweden	12	11	0.917 (0.646, 0.996)	HT1, HT2
Latvia	11	5	0.455 (0.213, 0.720)	HT2
Finland	5	3	0.6 (0.231, 0.882)	HT1, HT2
USA	40	0	0 (0, 0.087)	HT1

All statistical analyses were performed in R v. 4.01 (R Core Team 2020). Assuming a binomial distribution for the infection frequency (*p*), we estimated 95% confidence intervals for *p* for each population using the “binconf” function in the package *Hmisc* (Harrell and Dupont 2018). Finally, we performed Fisher's exact tests to determine if pairwise differences in infection frequency exist among *R. cerasi* populations spatially and/or temporally across the fly's native range.

### 2.2 Spatial Interpolation of the *w*Cer2 Infection Frequency in the Native Range

To infer the region(s) in Europe from which *R. cerasi* may have been introduced, we first captured spatial variation in *w*Cer2 frequencies by inferring distance‐dependent population variance of the *w*Cer2 infection frequency in the native range using field estimates of *w*Cer2 infection frequencies gathered over 25 years (Riegler and Stauffer 2002; Arthofer et al. 2009; Schuler et al. 2016; Bakovic et al. 2018). We complemented this dataset with *w*Cer2 infection frequencies from the 14 additional populations sampled for this study (Table S1). The raw dataset consisted of 270 populations sampled from 24 countries. A total of 2911 individuals were genotyped in the native range with a mean per population in the native range was 12.2 (range = 1–68). In the introduced range, we genotyped 40 individuals (Table S2). The estimates of variance for each population are based on independent samples (Wagner et al. 2005), therefore we randomly filtered locations sampled twice, leaving 238 populations for the downstream analysis (Figure 1B).

Next, to estimate distance‐dependent population variance which accounts for spatial autocorrelation (Wagner 2003), we applied variogram analysis, a method robust to sampling variance (Guillot et al. 2009). We fitted the variogram for a single locus (*wsp*) and analyzed *w*Cer2 infection frequencies obtained for each population. A single locus variogram can be interpreted as the proportion of unlike links against distance (Wagner et al. 2005), giving a range at which statistical dependence disappears (Guillot et al. 2009). First, we computed an empirical semivariogram by the “variogram” function using the *gstat* package in R (Pebesma 2004) and modelled autocorrelation structure by fitting a theoretical variogram (Wagner et al. 2005). The variogram map and empirical variograms fitted for different isotropy axes confirmed a lack of anisotropy in our data (see Figure S1 and Supporting Information for more details). Therefore, we fit an exponential theoretical variogram to model autocorrelation due to stationary spatial processes following:

$$\gamma \left(r\right)=C_{0}+C_{1}\left[1-\exp\left(\frac{-3r}{b}\right)\right]$$

where $\gamma \left(r\right)$ is a measure of dissimilarity between pairs of observations and $C_{0}$ is the proportion of variance that is not spatially structured and $C_{1}$ is the spatially structured variance component, providing an estimate of the population variance while accounting for spatial autocorrelation (Wagner et al. 2005).

### 2.3 Predicting Infection Frequencies in Unsampled Regions Across the Native Range

To predict infection frequency in unsampled regions across the *R. cerasi* native range, we performed spatial interpolation of the *w*Cer2 infection frequency. We first created a grid of coordinates using the *sf* package in R (Pebesma 2018). Spatial interpolation was then computed using the “krige” function in the *gstat* package in R. Here, kriging utilizes the fitted theoretical variogram to interpolate values $z\left(x_{i}+r_{i}\right)$ at any unobserved location based on a distance–variance relationship from the observed location, $x_{i}$ returning pairwise prediction values (Table S3). We then applied ordinary kriging with a constant intercept, *z* ~ 1, where *z* is the infection frequency of the *w*Cer2, and validated the model (see Supporting Information for more details). The variogram analysis, aided by our 25 year dataset, coupled with predictions of infection frequency in unsampled regions, allows us to infer the region(s) in Europe from which the introduced population of *R. cerasi* in the United States may have originated.

## 3 Results

### 3.1 *Rhagoletis cerasi* Lacks the *w*Cer2 Strain in the Introduced Range

The 40 *R. cerasi* sampled from the introduced population located in Niagara County, New York in the northeastern United States were uninfected with the *w*Cer2 strain (*p* = 0 [0, 0.08]) (Figure 1A; Table 1). The field estimate of *p* was produced from sampling several locations within the continuous area of outbreak in Niagara County and are, therefore, representative of the region and considered a single population. In accordance with previous studies, the 40 individuals uninfected with the *w*Cer2 strain were associated with the HT1 mitochondrial haplotype (Table 1) (Schuler et al. 2016; Schebeck et al. 2019).

### 3.2 *w*Cer2 Infection Frequencies Vary Spatially Across the Native Range

Our field estimates of *p* showed fixation of *w*Cer2 in areas of southwestern and Central Europe, which were associated with HT2 mitochondrial haplotype (Figure 1B; Table 1). One population is southwestern France located in Bayont (France 2, Table 1) showed a *w*Cer2 infection frequency of 83.3% (*p* = 0.83 [0.552, 0.953]), but was not significantly different from the other three French populations (Fisher's exact test: *P* = 0.478). The same French population from Bayont, though *w*Cer2 infected in high frequency, was exclusively associated with HT1 (Table 1). Populations in Turkey and Greece in the eastern portion of the range were *w*Cer2 uninfected and were associated with HT1. We did not detect a single *w*Cer2‐uninfected fly associated with the HT2 haplotype. The estimates of infection for population in Poland, Sweden, and Finland varied between *p* = 0.6 and *p* = 0.917, where some *w*Cer2‐infected individuals were associated with HT1 instead of HT2. The easternmost population in Russia showed low *w*Cer2 infection frequency (*p* = 0.167 [0.0470, 0.448]).

**FIGURE 2:**
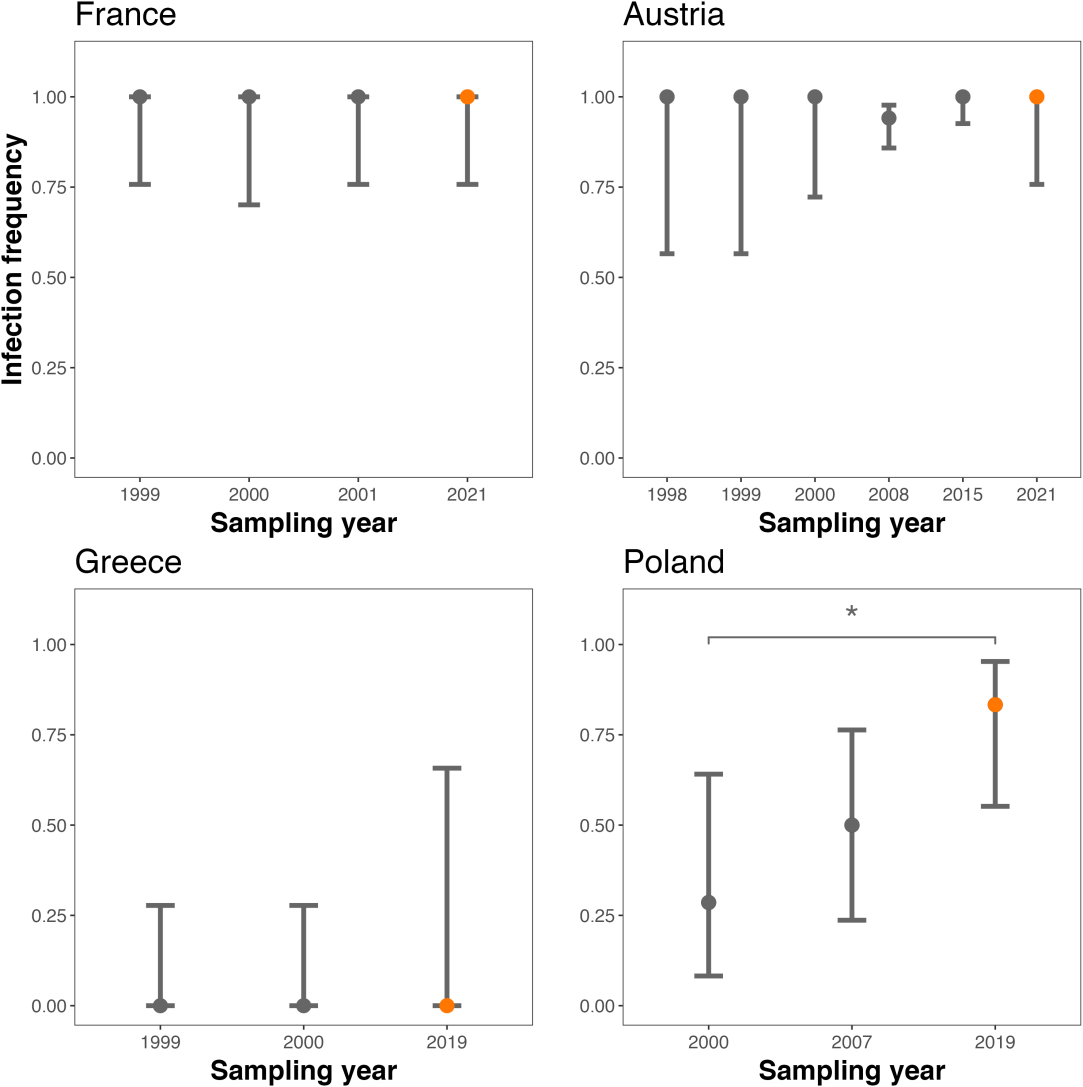
The *w*Cer2 infection frequencies for different regions of the native *Rhagoletis cerasi* range. Data from the present study (orange point) are compared to prior field estimates. Error bars represent 95% binomial confidence intervals. An asterisk indicates a statistically significant difference between the present study and prior years at *P* < 0.05.

To test for temporal variation in the *w*Cer2 frequencies, we compared infection frequencies of population sampled for this study to prior filed estimates of *p* across sampling years (Figure 2). Our pooled *p* estimate of French populations from this study was not significantly different from those of pooled regional estimates from 1999 (*p* = 1; Fisher's exact test: *P* = 1), 2000 (*p* = 1; Fisher's exact test: *P* = 1) and 2001 (*p* = 1; Fisher's exact test: *P* = 1). Resampling of the population in Austria showed no significant differences compared to previous sampling years (*p* = 1; Fisher's exact test: *P* = 1). The population in Greece uninfected in 1999 remained uninfected in 2000 and 2019, though with large credible intervals due to low sampling size (*p* = 0 [0, 0.658]). The populations sampled from Poland increased significantly in frequency from 2000 to 2019 (*p* = 0.286; Fisher's exact test: *P* = 0.045), but not from 2000 to 2007 (*p* = 0.5; Fisher's exact test: *P* = 0.172). Overall, this analysis revealed a general lack of temporal variation of the uninfected and fixed *w*Cer2 populations (Figure 2). Together, these results enabled us to pool all field estimates of the *w*Cer2 infection frequencies over the last 25 years and perform a spatial population prediction of infection frequencies in unobserved locations.

### 3.3 Identifying the *w*Cer2‐Uninfected Native Regions as Likely Candidates for Invasion Origin

**FIGURE 3:**
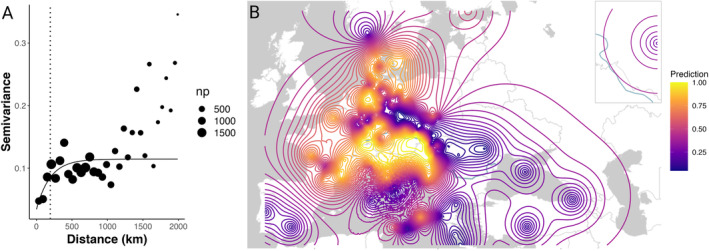
Spatial population variance and interpolation of the *w*Cer2 infection frequency in *Rhagoletis cerasi* native range predicting the regions from which North American populations of *R. cerasi* were introduced. (A) Semivariogram of the *w*Cer2 infection frequency. The solid line indicates the fitted exponential variogram model. The dashed vertical line indicates where the curve reaches 95% of the estimated population variance. Data points are shown with a spatial lag distance of 60 km. The size of each point represents the number of point pairs whose separation in the lag interval was used to estimate the semivariance. (B) Spatial interpolation represented with contour lines showing the predicted *w*Cer2 infection frequencies across the native range from 0% infection (dark blue) to 100% infection (yellow), including Russia (inset).

To assess the spatial structure of *w*Cer2 in the native range, we computed distance‐dependent population variance (Figure 3A). The data points of the variogram were grouped into classes over the whole sampling range (0–6300 km) with a distance interval of 60 km. The semivariance first progressively increased up to the distance of about 200 km, after which it plateaued. For scales below 1000 km, the infection frequency was strongly spatially autocorrelated. After the cut‐off distance of 2000 km, additional information did not improve variance estimates.

Results of the spatial interpolation analysis of infection frequencies predicted fixation of the *w*Cer2 in *R. cerasi* populations from Central and Western Europe (Figure 3B) with high confidence (prediction variance ranged from 0.04 to 0.06) (Figure S2; Table S3) due to high sampling density in these regions. The Mediterranean and the eastern portions of the fly's native range including Eastern Europe and Turkey were predicted to be *w*Cer2 uninfected (Figure 3B) with a prediction variance of below 10% (Figure S2; Table S3).

## 4 Discussion

We investigated the invasion of the European cherry fruit fly *R. cerasi* in North America using *Wolbachia* infection frequencies in the fly's native range as a marker to reconstruct the area of invasion origin. Our estimates of *w*Cer2 infection frequencies (*p*) showed that the introduced *R. cerasi* population in the northeastern United States did not carry *w*Cer2 and the associated mitochondrial haplotype suggesting a founder effect from *w*Cer2‐uninfected native populations. Field estimates of the *w*Cer2 infection frequency in the *R. cerasi* native range, combined with the field estimates of *p* spanning more than 238 populations sampled across 25 years, allowed us to assess distance‐dependent population variance, predict infection frequency in unsampled regions and rule out Central and Western Europe as likely source populations. Our results implicate a number of narrow regions in the Mediterranean and the eastern Europe as the most likely areas from which the flies were introduced into North America (Figure 3B). Our findings provide invaluable information about endosymbiont diversity in the introduced flies, shed light on potential source populations, and highlight that *Wolbachia* endosymbionts can be used as a tool to infer insect invasion origins.

The *w*Cer2 infection frequencies varied spatially across the *R. cerasi* native range (Figure 1A; Table 1). We documented the fixation of the *w*Cer2 in Central and Western Europe regions while observing a lack of the *w*Cer2 in Eastern Europe and the Mediterranean (Figure 1A; Table 1). In addition, we observed a lack of temporal variation in infection frequencies across years (Figure 2) in *w*Cer2 uninfected or fixed regions, which appeared to be stable over time. An exception from temporal stability among the fixed populations in Austria was the pooled *p* estimate from 2008 showing infection frequency less than 100%, though not significantly different from 1998 to 2000, 2015 and 2021 (Figure 2). We acknowledge temporal variation in this region may be due to low *Wolbachia* titer at concentrations below the detection threshold. However, low densities of *Wolbachia* have been associated with alternative reproductive strategies of the endosymbiont (Ijichi et al. 2002), often resulting in a lack of CI induction. We observe strong levels of CI in the *w*Cer2 strain, however, suggesting that low titer is unlikely responsible for the pattern. In *Drosophila*, both environmental factors and/or a variation in maternal transmission have been hypothesized to play a role in temporal fluctuations in *Wolbachia* infection frequencies (Turelli and Hoffmann 1995; Hague et al. 2020).

Similarly, the 2019 population from Greece harboring only two *w*Cer2‐uninfected individuals had large binomial confidence intervals, but was not significantly different from the two other *w*Cer2‐uninfected Greek populations. Due to low sampling size, it is possible that we failed to detect infection in the 2019 population. However, this scenario is unlikely considering a vast uninfected area surrounding the Greek populations (Figure 2). Our estimate of *p* for the population from Poland in 2019 was significantly higher compared to the pooled estimate from 2000 (Figure 2). This pattern supports the hypothesis of ongoing spread of the *w*Cer2 in *R. cerasi* populations in central Europe previously documented by Riegler and Stauffer (2002), Schuler et al. (2016) and quantified by Bakovic et al. (2018). Including the *w*Cer2 infection frequencies in the areas of active spread across the fly's native range further allowed us to “fine tune” the spatial interpolation analysis. Together, the pattern of spatial variation of the *w*Cer2 infection frequencies across the *R. cerasi* native range and a general temporal stability of the uninfected and fixed *w*Cer2 populations (Figure 2) enabled us to pool all field estimates of the *w*Cer2 infection frequencies over the last 25 years to predict infection frequencies in unobserved locations.

Based on the spatial interpolation prediction of the *w*Cer2 infection frequency, we argue that *R. cerasi* unlikely originated from Central and Western Europe where fly populations are either fixed for *w*Cer2 or are infected at very high frequencies (Figure 3). A deviation from this pattern is a narrow belt in central Germany (Figure 3B), accounting for a mosaic pattern of the *w*Cer2 infection in this region (Schuler et al. 2016; Schebeck et al. 2019). We propose regions that harbor *w*Cer2‐uninfected individuals as the most likely candidates for invasion source, originating from Eastern Europe and the Mediterranean region. Concurrently, field estimates and spatial interpolation analysis, which take into account sampled regions and predict infection frequencies at unsampled locations, showed that these regions lack the *w*Cer2 strain and could have served as source population(s) (Figure 1A; Figure 3; Table 1). These populations were also associated with HT1 found in *R. cerasi* in the introduced range (Table 1), strongly associated with the fixed *w*Cer1 strain (Schuler et al. 2016; Schebeck et al. 2019).

In Bakovic et al. (2018), the authors estimated the speed of the *w*Cer2 spatial spread in the native range of *R. cerasi*. However, the nature of *w*Cer2's local dynamics and spatial spread remains unknown. The mode of *Wolbachia* local dynamics in natural populations can take two forms depending on the value of effective fecundity. If F (1–*μ*) < 1, as in *Aedes aegypti* mosquitoes, *Wolbachia* will stabilize at 0, when present at very low frequency, following the bistable local dynamics (Hoffmann et al. 2014). Conversely, when F (1–*μ*) > 1, exemplified by the *w*Ri strain in *D. simulans*, even very low numbers of *Wolbachia* are sufficient to push infection frequency to high stable equilibrium following Fisherian dynamics (Hoffmann, Turelli, and Harshman 1990; Barton and Turelli 2011). If we assume that *w*Cer2 infection follows bistable local dynamics, the fact that we failed to detect the presence of the HT2 mitochondrial haplotype associated with the *w*Cer2 strain in the introduced range strongly suggests that *w*Cer2 was unlikely from an infected native population in low numbers that stabilized at *p* = 0 at the time of sampling. If, however, *w*Cer2 dynamics behave according to Fisherian dynamics, even invading in low initial numbers in the introduced range would have driven *w*Cer2 to high frequencies, unless impeded by differing selection pressures in the newly invaded habitat similar to *Wolbachia* in the Argentine ant, *Linepithema humile* (Reuter, Pedersen, and Keller 2005). In the latter case, given the strong association between *w*Cer2 and HT2, we would expect to detect this mitochondrial haplotype in the invasive range as a remnant of such an event. Taken together, evidence strongly supports a founder effect scenario where native *w*Cer2‐uninfected flies served as the founding individuals.

We did detect deviations from *w*Cer2/HT2 association in the native range, namely in transitional populations from France, Poland, Sweden, and Finland, where field estimates of *p* varied between 0.60 and 0.917. Here, some *w*Cer2‐infected individuals were associated with HT1 (Table 1). Deviations from this association were highlighted in Schuler et al. (2016) who detected *w*Cer2/HT1 co‐occurrence in transitional German populations with a frequency of 21.9% (Schebeck et al. 2019). The incidence of the association between *w*Cer2 and HT1 could be the result of either paternal (e.g. Hoffmann and Turelli 1988) or intraspecific horizontal transmission (Schuler et al. 2016). Using mathematical modeling of *Wolbachia* and mitochondrial haplotype dynamics, Schuler et al. (2016) showed that the association of *w*Cer2 with HT1 is transient, likely due to weak maternal inheritance of *w*Cer2 and will be lost after complete invasion of *w*Cer2. Their analysis also showed that this association, if transmitted only somatically, would be lost after fixation of the maternally transmitted *w*Cer2. These low levels of misassociation between the *Wolbachia* strain infecting the fly and fly's mitochondrial haplotype in the native range suggest that the chances that the invasion originated from *w*Cer2/HT1 individuals in transitional populations, followed by a subsequent loss of *w*Cer2, are low. There is a possibility that *w*Cer2 is present in the introduced range, but remained undetected in our sample. We find this scenario unlikely considering a large sample size (*n* = 40 individuals) along with the fact that our sampling method included several locations within the continuous area of in Niagara County, therefore providing a solid representation of the invaded region.

Our current data lack the sensitivity to detect multiple invasion origins of *R. cerasi* into North America. Many successful biological invasions are accomplished through multiple introductions to the extent that single introductions are often considered exceptions (Dlugosch et al. 2015; Caizergues et al. 2024). Recurrent introductions can alleviate the adverse effects of demographic bottlenecks associated with founder effects and facilitate the establishment of invasive species in the new range (Verhoeven et al. 2010). However, negative impacts of multiple sources of introduction have also been observed when gene flow between genetically distinct source populations in the invasive range leads to the breakdown of locally adapted genotypes (Rius and Darling 2014; Barker et al. 2019). Future studies will aim to incorporate population‐level genomic variation of the flies across their natural range to corroborate our *Wolbachia* results.

In a similar study, Zhang et al. (2014) used the obligatory mutualistic endosymbiont *Buchnera aphidicola* to trace invasion routes in the clonal Russian wheat aphid. A small number of studies have also used *Wolbachia* to infer demographic history during colonization such as the Argentine ant, *Linepithema humile* (Reuter, Pedersen, and Keller 2005), and fire ants (*Solenopsis* spp.) (Shoemaker et al. 2000). This study also utilizes *Wolbachia* as a marker to focus on the question of invasion origin of *R. cerasi* in North America, but achieves a high resolution by leveraging an extensive and thorough range‐wide sampling of the native range. In contrast, classic population genetic markers used in invasion biology studies include microsatellites, and mtDNA that often have limited resolution to detect the origin of the invasion (Zhang et al. 2014). In insects, the interpretation of mitochondria‐based biogeographic and colonization history analysis is confounded due to the linkage with maternally transmitted endosymbionts (Hurst and Jiggins 2005; Arif et al. 2021). Our study confirms that when used together, *Wolbachia* and mtDNA markers can shed light on demographic histories and invasion processes while representing an inexpensive and rapid alternative if genomic data are not available at hand. Given that increasing number of studies report spatial and temporal patterns of *Wolbachia* infection frequencies (Hoffmann and Turelli 1988; Shoemaker et al. 2003; Kriesner et al. 2016; Hague et al. 2020) and a high prevalence of *Wolbachia* in insects (Werren and Windsor 2000; Jiggins et al. 2001), as well as other arthropods and nematodes (Weinert et al. 2015), the use of spatially varying *Wolbachia*‐based markers open up a promising avenue in elucidating invasion origins and a better understanding of the factors governing introductions. Additionally, markers such as SNPs and structural variants that allow researchers to include within‐strain *Wolbachia* variation (Wolfe et al. 2021) could heighten the resolution and help flesh out invasion scenarios at an even finer scale.

## 5 Conclusion

Our sampling of the *R. cerasi* introduced range revealed that the flies lacked the *w*Cer2 strain and the associated mitochondrial haplotype. Our estimates of *w*Cer2 infection frequency varied spatially across the native range. The extensive sampling on the *R. cerasi* native region enabled us to quantify population variance in infection frequencies, rule out Central and Western Europe of *R. cerasi* range as the source population, and propose *w*Cer2‐uninfected *R. cerasi* populations from Eastern Europe and the Mediterranean region as the most likely candidates for the invasion origin. Our study highlights the utility of spatially varying *Wolbachia* frequencies to infer species invasion origins and demographic histories. More broadly, it puts forward endosymbionts as powerful tools to reconstruct invasion histories.

## Conflicts of Interest

The authors declare no conflicts of interest.

## Supporting information


Appendix S1.

Appendix S2.

Appendix S3.

## Data Availability

The raw data are available in the supplemental data files. The R code used for statistical analysis, spatial modeling, and constructing the figures can be found in the following link: https://github.com/slecic/USA_invasion_paper.
