# Supplementary material for: Spatially Varying Wolbachia Frequencies Reveal the Invasion Origin of an Agricultural Pest Recently Introduced From Europe to North America
Source: Evol Appl. 2024 Sep 20;17(9):e70016. doi: 10.1111/eva.70016 (PMC11413411; doi:10.1111/eva.70016)
Supplement: Supplementary file 1 — Appendix S1. [file EVA-17-e70016-s002.docx]

**Supplemental Information for ‘Spatially varying *Wolbachia* frequencies reveal the invasion origin of an agricultural pest recently introduced from Europe to North America’**

**Supplemental Methods**

To determine if pattern of spatial variation of the *w*Cer2 infection frequency violates the assumption of weak stationarity, we tested for the presence of anisotropy (directionality) (Suppl. Fig. 1A, 1B). If the assumption of stationarity is not violated, the structure of autocorrelation stays the same in all directions across the studied range, with a constant mean and variance. In this case, autocorrelation depends only on the geographic distance between populations (Wagner et al. 2005).

To predict infection frequency in unsampled regions across the *R. cerasi* native range, we applied ordinary kriging with a constant intercept, z ~ 1, where z is the infection frequency of the *w*Cer2 strain. In addition to plotting the prediction of the *w*Cer2 infection frequency (Fig. 3B), we also plotted the prediction variance (Suppl. Fig. 2). Finally, we validated the model using n-fold cross-validation incorporated in gstat.cv function in the *sf* package in R (R Core Team 2020). This method partitions the data set into a specified number of parts. Predictions for all observations in a defined part are made based on the remaining N-1 parts, and this is repeated for each of the N parts of validation. Therefore, we define the N-fold parameter for all observations (rows) of the dataset. Parameter nmax, the number of nearest observations, was set to match the width parameter of the experimental variogram and the maxdist parameter was similarly set to match the range value in the theoretical variogram. The mean residual error was close to 0 (0.01104178), with a low mean square predicted error (MSPE) of 0.1170851, and a mean square normalized error close to 1 (0.7057565). We found a high correlation between the observed and predicted values (0.7015238) and a low correlation between the predicted values and residuals (0.2336373), which is desirable.

**Literature Cited**

R Core Team. (2020). *R: A language and environment for statistical computing*. R Foundation for Statistical Computing, Vienna, Austria.

Wagner, H. H., Holderegger, R., Werth, S., Gugerli, F., Hoebee, S. E., & Scheidegger, C. (2005). Variogram analysis of the spatial genetic structure of continuous populations using multilocus microsatellite data. *Genetics*, *169*(3), 1739–1752. <https://doi.org/10.1534/genetics.104.036038>

**Supplemental Table 1.** The locations of the introduced and native *R.* *cerasi* populations including GPS coordinates, year of collection and collection dates of each site.

| **Country** | **Location** | **Latitude/Longitude** | **Collection Year** | **N** |
| --- | --- | --- | --- | --- |
| Austria | Vienna | 48.288306/16.427444 | 2021 | 12 |
| Italy | Monza | 45.583332/ 9.266667 | 2021 | 12 |
| France 1 | Lyon | 45.88288964913/4.793641654542 | 2021 | 12 |
| France 2 | Byant | 45.18029750810/1.68972617493 | 2021 | 12 |
| France 3 | Tours | 46.3541257518/-0.538824983260 | 2021 | 12 |
| France 4 | Arras | 50.3964283762/3.045462229679 | 2021 | 12 |
| Turkey 1 | Kemehisar/Merkez/Niğde | 37.8104224/ 34.4930186 | 2019 | 9 |
| Turkey 2 | Salkımlı/Artvin | 41.1959834/ 41.8726167 | 2019 | 10 |
| Russia | Tomsk | 56.50015455476/84.9861328407 | 2019 | 12 |
| Greece | Kamarina | 39.13333/20.666664 | 2019 | 2 |
| Poland | Kraków | 50.06003514810/19.94668855430 | 2019 | 12 |
| Sweden | Österslöv | 56.11625699983/14.26450699883 | 2019 | 12 |
| Latvia | Institute of horticulture | 56.57914588563/23.37308088056 | 2019 | 11 |
| Finland | Helsinki | 60.17458411842/24.94786022621 | 2022 | 5 |
| United States | New York/Niagara County | 42.3507/ -78.18901 | 2018 | 40 |


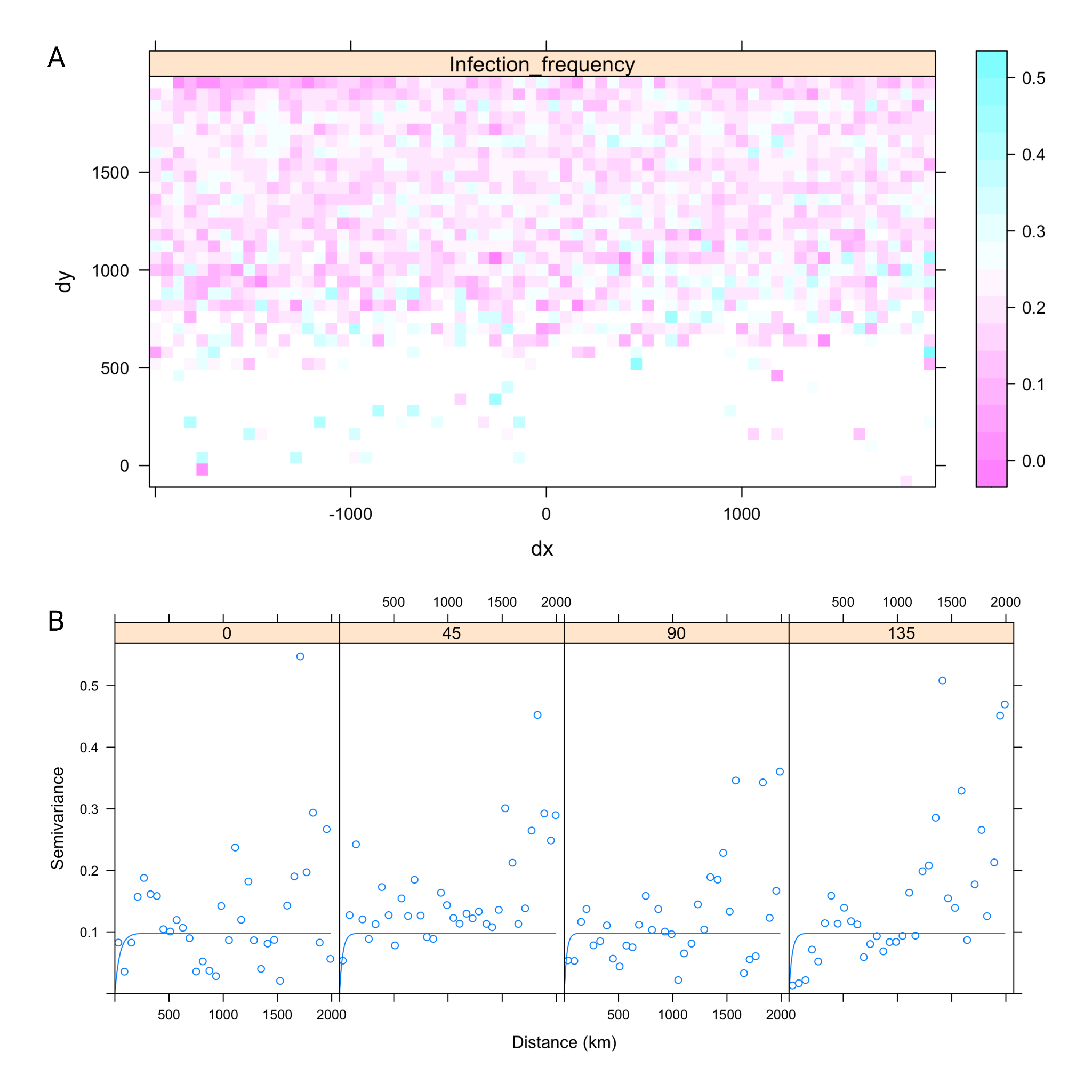


**Supplemental Figure 1.** Structure of the autocorrelation between *w*Cer2 infection frequencies across the native range for (A) for the whole region considered in the variogram analysis, and (B) for directions of North (0), Northeast (45), East (90), and Southwest (135). The lack of directionality and the presence of constant variance is confirmed by the presence of a sill (i.e., all theoretical fit curves have a plateau).


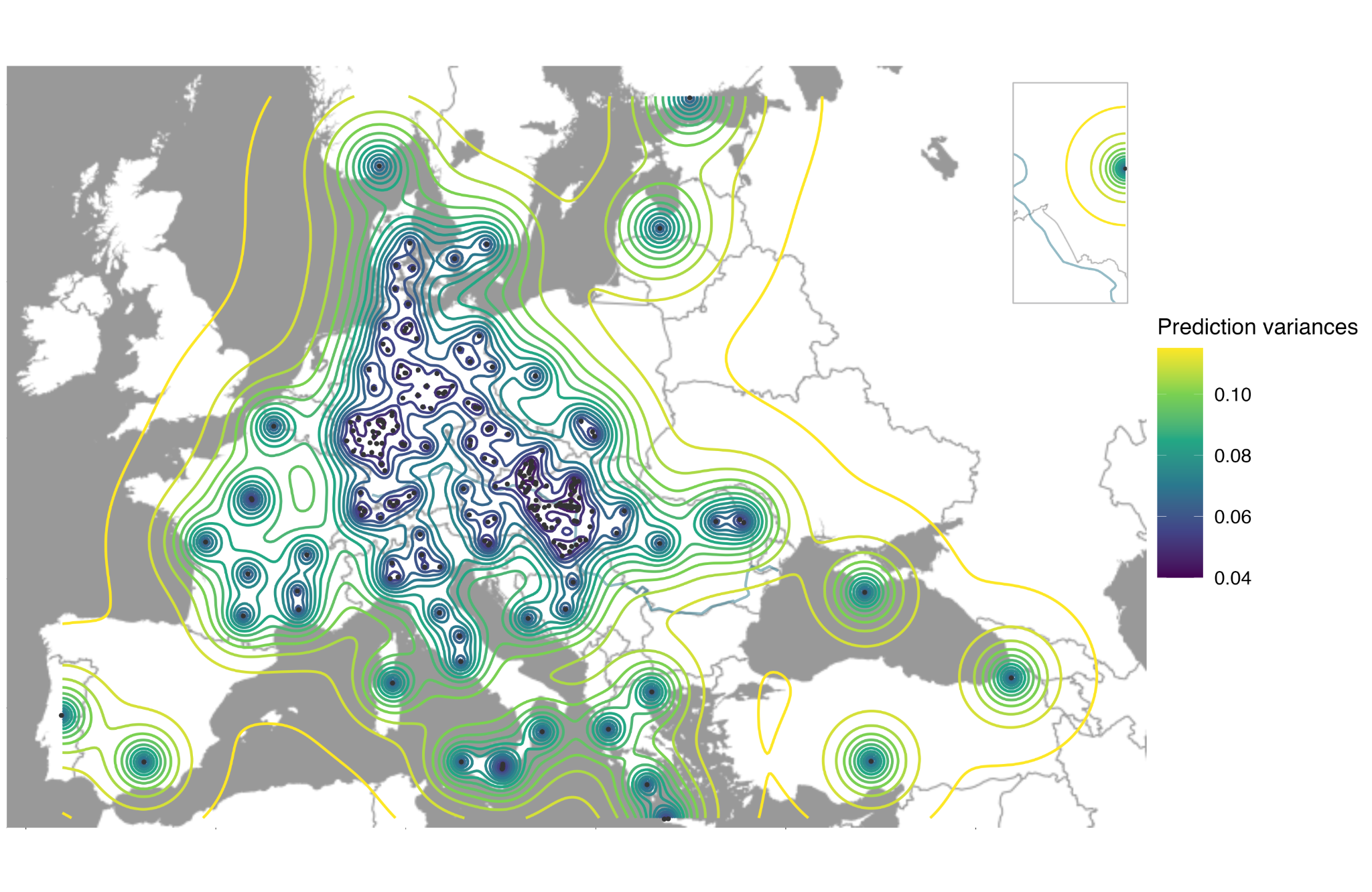


**Supplemental Figure 2.** The prediction variance of the *w*Cer2 infection frequencies across the native range shown in Figure 3B. The contour lines are overlaid on a geographic map to indicate regions with low prediction variance in dark blue and high prediction variance in yellow. Prediction variance for the regions surrounding the population from Russia is inserted in the upper right corner. In total, 238 populations across 24 countries in the native fly range are used (see Figure 1B for distribution of sampling locations in the fly’s native range).
